# Metabolomic Profiling Reveals Biomarkers in Coronary Heart Disease Comorbidity

**DOI:** 10.1155/jdr/8559677

**Published:** 2024-12-19

**Authors:** Chunmei Geng, Benhui Liang, Zihan Kong, Lei Feng, Jianhua Wang, Qingying Si, Pei Jiang

**Affiliations:** ^1^Department of Pharmacy, Tianjin First Central Hospital, School of Medicine, Nankai University, Tianjin, China; ^2^Department of Cardiology, Xiangya Hospital, Central South University, Changsha, Hunan, China; ^3^Translational Pharmaceutical Laboratory, Jining First People's Hospital, Shandong First Medical University, Jining, Shandong, China; ^4^Department of Neurosurgery, Jining First People's Hospital, Shandong First Medical University, Jining, Shandong, China; ^5^Department of Endocrinology, Tengzhou Central People's Hospital, Tengzhou, Shandong, China

**Keywords:** comorbidities, coronary heart disease, gas chromatography, mass spectrometry, metabolomics, multivariate statistical analysis

## Abstract

**Background and Aims:** Coronary heart disease (CHD), hypertension (HTN), depression (Dep), and Type 2 diabetes mellitus (T2DM) are often comorbid, resulting in an exacerbated patient condition and worsened prognosis. A lack of systematic metabolomic studies on comorbidities of CHD remains. Therefore, comprehensive metabolomic-based evaluation of comorbidities of CHD is necessary.

**Methods and Results:** In the current study, 169 healthy subjects, 149 CHD subjects, 107 CHD + HTN subjects, 126 CHD + Dep subjects, and 58 CHD + T2DM subjects were recruited. Gas chromatography–mass spectrometry was used for metabolite determination, and multivariate statistical analysis was conducted to identify metabolites that are differentially expressed with the comorbidities of CHD. There were 9, 16, 14, and 10 metabolites identified in the healthy and CHD group, the CHD and CHD + HTN group, the CHD and CHD + Dep group, and the CHD and CHD + T2DM group, respectively. Six metabolic pathways were affected, involving starch and sucrose metabolism; fructose and mannose metabolism; citrate cycle; alanine, aspartate, and glutamate metabolism; fatty acid biosynthesis; and glycolysis.

**Conclusion:** Our study has systematically elucidated the metabolic changes underlying the comorbidities of CHD, thereby providing insight into the mechanisms associated with these alterations.

## 1. Introduction

Despite a decline in coronary heart disease (CHD) incidence in most countries, CHD remains the leading cause of mortality worldwide. In addition, hypertension (HTN), depression (Dep), and Type 2 diabetes mellitus (T2DM) are often comorbid with CHD and can induce or worsen the development of CHD and affect the prognosis [1–4]. Although previous studies have been done on CHD, HTN-comorbid CHD, Dep-comorbid CHD, and T2DM-comorbid CHD and many risk factors including genetic and environmental factors have been proposed [2, 5–11], the underlying mechanisms cannot be fully explained by these risk factors. Thus, a novel approach to assess the comprehensive metabolic status in comorbidities of CHD is needed.

Metabolomics is a rapidly evolving field of life science that uses advanced analytical chemistry techniques in conjunction with univariate and multivariate statistical analyses to systematically characterize the metabolome [12]. Our previous metabolomic studies based on liquid chromatography–mass spectrometry (LC–MS) and gas chromatography–mass spectrometry (GC–MS) identified many metabolites and revealed changes in metabolites from a global perspective, contributing to the understanding of the underlying mechanisms [13–16]. Metabolomic studies were conducted on CHD, HTN, Dep, or T2DM, but the overlapping metabolic disturbances in these comorbidities of CHD were insufficiently described. Therefore, studies designed to fully understand the metabolic signatures of these comorbidities of CHD are urgently needed.

In our study, we sought to elucidate the metabolic signatures of these comorbidities of CHD. To this end, a GC–MS-based metabolomic approach combined with sophisticated statistical methods was employed to identify metabolic biomarkers in CHD, HTN-comorbid CHD, Dep-comorbid CHD, and T2DM-comorbid CHD. The results could provide an objective diagnostic method and help to identify metabolomic signatures that better identify risk groups early, as well as improve our understanding of pathophysiologic pathways.

## 2. Methods

### 2.1. Subjects

Patients were recruited at the outpatient clinic of Jining First People's Hospital in Jining, China. The diagnosis of CHD was made by at least two experienced cardiologists and confirmed using coronary angiography results (significant coronary artery stenosis ≥ 50% in at least one of the three major coronary arteries or major branches). Among all the patients, 149 had CHD with no HTN, Dep, or T2DM; 107 had CHD comorbid with HTN defined as systolic blood pressure ≥ 140 mmHg or diastolic blood pressure ≥ 90 mmHg, a history of HTN, or current antihypertensive treatment; and 126 had CHD comorbid with Dep assessed by at least two experienced psychiatrists according to the fifth edition of the *Diagnostic and Statistical Manual of Mental Disorders* criteria for major depressive disorder, which is characterized by significantly depressed mood and anhedonia. The severity of depressive symptoms was scored with the Patient Health Questionnaire-9, a nine-item questionnaire that is commonly used in outpatient settings. The scale uses a cutoff score for Dep analysis of ≥ 5. The last group of patients included 58 who had CHD comorbid with T2DM. T2DM was diagnosed based on one of the following criteria set by the World Health Organization: a fasting plasma glucose (PG) ≥ 7.0 mmol/L, a 2-h PG after a 75-g glucose load ≥ 11.1 mmol/L, an HbA1c ≥ 6.5%, or receiving antidiabetic medication. In addition, the 169 healthy controls, who were frequency matched for age and sex, were adults without CHD, HTN, Dep, or T2DM. These individuals had undergone a series of assessments, including clinical physical examination, radiographic chest examination, electrocardiogram, and evaluation of their medical history. Those with cancer, severe autoimmune disease, or severe liver and/or kidney disease were excluded. The demographic and clinical characteristics of the study subjects, including age, sex, height, weight, smoking status, and drinking habits, were obtained from medical record systems and questionnaires. In our study, smoking and drinking were defined as follows: A person with a smoking history is defined as someone who has smoked 100 or more cigarettes in their lifetime and has used any type of smoked tobacco product within the past 30 days, either on a daily basis or occasionally [17]. A person with a drinking history is defined as someone who has consumed ethanol in the form of alcohol, amounting to 14 g or more per occasion, on a weekly basis or more frequently, over a period of more than 6 months [18, 19]. Our study was approved by the Ethical Review Board of Jining First People's Hospital (No. JNRM-2021-LC-104). All subjects provided written informed consent to allow use of their samples for analysis and data sharing.

### 2.2. Reagents and Instruments

Heptadecanoic acid (purity: ≥ 98%), an internal standard (IS), and N,O-bis(trimethylsilyl)trifluoroacetamide with 1% of trimethylchlorosilane (BSTFA + 1% TMCS) (*v*/*v*) were obtained from Sigma-Aldrich (Saint Louis, Missouri, United States). Pyridine was purchased from Shanghai Macklin Biochemical (Shanghai, China). O-Methyl hydroxylamine hydrochloride (purity: 98.0%) was obtained from J&K Scientific (Beijing, China). Chromatographic-grade methanol was obtained from Thermo Fisher Scientific (Waltham, Massachusetts, United States). Water was purchased from Hangzhou Wahaha Company (Hangzhou, China). The 7890B GC system with a 7000C triple–quadruple mass spectrometer, electrospray ionization (ESI), and HP-5MS fused-silica capillary column (30 m × 0.25 mm × 0.25 *μ*m) were from Agilent Technologies (Santa Clara, California, United States).

### 2.3. Sample Preparation

Blood samples from all subjects were collected during a fasting period to accurately reflect the metabolic profile. These samples were then subjected to centrifugation at 5000 rpm for 10 min at room temperature before being stored at −80°C until analysis. Serum samples (100 *μ*L) were mixed with 350 *μ*L methanol (containing 100 *μ*g/mL IS), vortexed (30 s), and centrifuged (14,000 rpm, 4°C, 10 min). The supernatant was transferred to a 2-mL tube and dried at 37°C under a gentle stream of nitrogen gas. The extracts were then mixed with 80 *μ*L of O-methyl hydroxylamine hydrochloride (15 mg/mL in pyridine) and incubated for 90 min at 70°C. Subsequently, BSTFA + 1% TMCS (100 *μ*L) was added to each sample, followed by incubation for 60 min at 70°C; the solution was vortexed, centrifuged (14,000 rpm, 4°C, 2 min), and filtered through a 0.22-*μ*m filter membrane before GC–MS analysis.

### 2.4. GC–MS Analysis

Sample analysis was conducted on a 7890B-7000C GC–MS (Agilent Technologies, California, United States). The specific operation conditions of chromatography were as follows: the chromatographic analysis was carried out with a HP-5MS capillary column, and the injection volume was 1 *μ*L with the split mode (50:1). The carrier gas was high-purity helium flowing at 1 mL/min. The temperature of injection was 280°C. The GC temperature program began at 60°C for 4 min, increased to 300°C at 8°C/min, and ended with a final 5-min maintenance at 300°C. Additionally, the conditions for mass spectrometry analysis were as follows: The ionization method was ISI, and the ion source temperature and the transfer line temperature were 230 and 250°C, respectively. The ionization voltage was 70 EV, and full scan mode was adopted with a quality scanning range of *m*/*z* 50–800. In general, 50 *μ*L of each serum sample from healthy subjects, CHD subjects, CHD + HTN subjects, CHD + Dep subjects, and CHD + T2DM subjects was obtained and then vortexed and mixed. Thus, this is the so-called quality control (QC) sample. Twenty-five samples based on the ratio of samples per group and five QCs were performed for 1 day (one batch), and 25 samples run randomized while the five QCs were distributed evenly among the 25 samples. At last, nine samples and five QCs were done. The peak area and retention time (RT) of the IS were applied to evaluate the stability of the instrument and the reproducibility of the entire analysis process.

### 2.5. Statistical Analyses

Demographic and clinical characteristics of the subjects were evaluated with the Student *t*-test (for normally distributed continuous variables), the Mann–Whitney test (for nonnormally distributed continuous variables), and logistic regression analysis. Categorical variables were displayed as numbers (percentage) of subjects within each group and were compared using the *χ*^2^ test. The above analyses were performed via SPSS 23.0 software (SPSS Inc, Chicago, Illinois, United States).

The primary GC–MS data were processed using Agilent Unknowns analysis software and MassHunter quantitative analysis software (Agilent Technologies, California, United States). This process enabled peak extraction; removal of peaks with a signal-to-noise ratio < 3; and peak deconvolution, alignment, and data reduction to produce a list of *m*/*z* and RT pairs, with the corresponding intensities for all detected peaks from each data file in the dataset [20, 21]. The resulting table was exported into Excel (Microsoft, Redmond, Washington, United States), and the normalized peak area percentages were used as the percentage of corresponding intensities of each peak/total peak area. Center scaling, unit variance scaling, and Pareto scaling are commonly used to perform the normalization data. In our study, we adopted the Pareto scaling. Too many missing values will cause difficulties for downstream analysis. There are several different methods for this purpose, such as replace by small values, mean/median, *k*-nearest neighbor (KNN), probabilistic principal component analysis (PPCA), Bayesian principal component analysis (BPCA) method, and singular value decomposition (SVD) method to impute the missing values [22, 23]. In our work, the default method replaces all the missing values with small values (the half of the minimum positive values in the original data) assuming to be the detection limit, and the data were not transformed. The resulting three-dimensional dataset including peak index (RT–*m*/*z* pairs), sample names (observations), and normalized peak area percentages was imported into SIMCA-P 14.0 (Umetrics, Umea, Sweden) for statistical analyses. SIMCA-P 14.0 software (Umetrics, Umea, Sweden) was used to perform principal component analysis (PCA), partial least squares-discriminant analysis (PLS-DA), and orthogonal partial least squares-discriminant analysis (OPLS-DA) of the healthy group and the CHD group, the CHD group and the CHD + HTN group, the CHD group and the CHD + Dep group, and the CHD group and the CHD + T2DM group, to select the significant variables that were responsible for group separation. *R*^2^*X*, *R*^2^*Y*, *Q*^2^*Y*, and variable importance in projection (VIP) values are important quality parameters for evaluating the performance of OPLS-DA models.

The two-tailed Student *t*-tests were performed using SPSS 23.0 to further test the differences between the two groups. Metabolites with VIP values > 1.0 in the OPLS-DA and *p* values < 0.05 in the two-tailed Student *t*-tests were considered potential discriminant metabolites. In the context of pathway analysis, libraries refer to the databases or sets of information used to match the metabolite data to known biological pathways. KEGG (Kyoto Encyclopedia of Genes and Genomes) and HMDB (Human Metabolome Database) are often applied. In our study, the finalized set of discriminant metabolites was imported into MetaboAnalyst 5.0 (http://www.metaboanalyst.ca) for metabolic pathway analysis, and pathways with *p* values < 0.1 and impact values > 0 were defined as significant, contributing to the biochemical interpretation of the metabolites. Venn diagrams are indeed a useful tool in the context of metabolomics, where comparing different groups helps identify common and unique metabolites. They can clearly show the relationships and intersections between different sets of data. Venn diagram analysis was conducted on the free OmicShare online platform (http://www.omicshare.com/tools). Details of the statistical analyses can be found in the supporting information (available here).

## 3. Results

### 3.1. Basic Characteristics of Subjects

A total of 169 healthy subjects, 149 CHD subjects, 107 CHD + HTN subjects, 126 CHD + Dep subjects, and 58 CHD + T2DM subjects were included in our study. The demographic and clinical characteristics of the subjects are indicated in Table 1.

There were no significant differences between the CHD and healthy groups in terms of age, body mass index (BMI), sex, smoking, or drinking (*p* > 0.05). Similarly, there were no significant differences in the basic characteristics between CHD patients with comorbid HTN, Dep, or T2DM and those with CHD alone (*p* > 0.05). Meanwhile, we included age, BMI, sex, smoking, and drinking to construct a multivariable logistic regression model. The results revealed no significant differences with respect to age, BMI, sex, smoking, or drinking among the following comparisons: CHD vs. healthy, CHD + Dep vs. CHD, CHD + HTN vs. CHD, and CHD + T2DM vs. CHD (*p* > 0.05) (see Supporting Information 2: Table S1).

### 3.2. GC–MS Total Ion Chromatograms of Serum Samples

**Figure 1:**
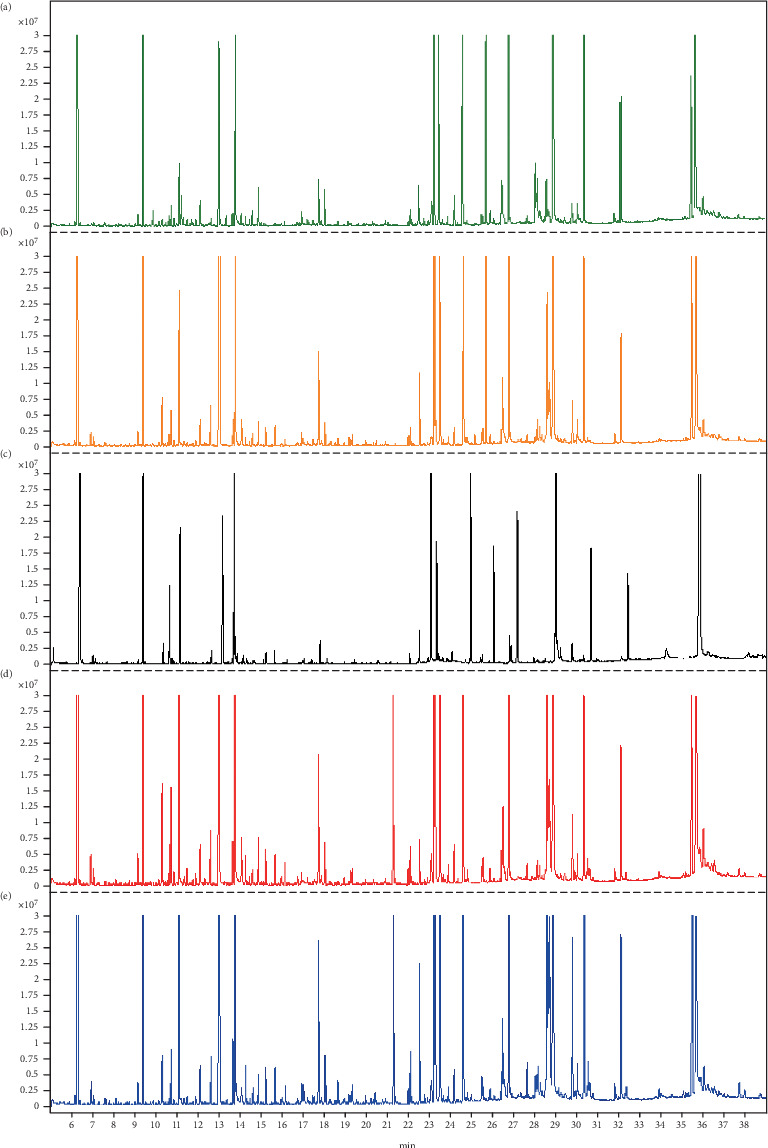
The representative gas chromatography–mass spectrometry (GC–MS) total ion chromatogram of the serum sample. (a) Healthy sample. (b) CHD sample. (c) CHD + HTN sample. (d) CHD + Dep sample. (e) CHD + T2DM sample.

The represented total ion chromatograms of serum samples (healthy, CHD, CHD + HTN, CHD + Dep, and CHD + T2DM) are shown in Figure 1. All samples had a strong signal response, and 114 metabolites were identified in each serum sample and then used in the subsequent multivariate analysis. In addition, the relative standard deviation (RSD) in intra- and interday of the peak area and RT of the IS were < 15%, indicating that the analytical instrument operated within acceptable standard variations.

### 3.3. Statistical Comparison of Metabolomic Data

**Figure 2:**
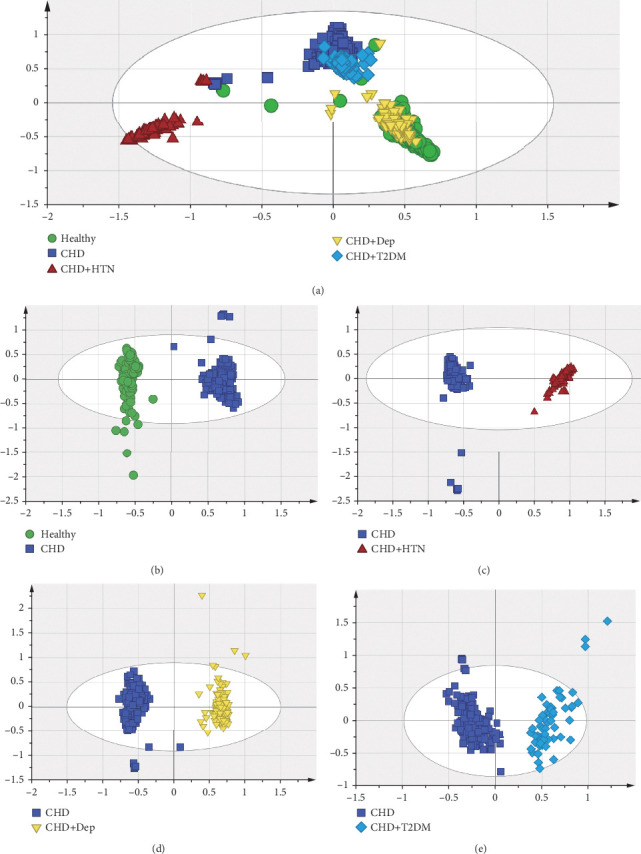
Orthogonal partial least squares-discriminant analysis (OPLS-DA) score chart. (a) Healthy, CHD, CHD + Dep, and CHD + T2DM group. (b) Healthy and CHD group. (c) CHD and CHD + HTN group. (d) CHD and CHD + Dep group. (e) CHD and CHD + T2DM group.

OPLS-DA was used to better identify discriminating metabolites that contributed to the classification of samples and to remove noncorrelated variations contained within the spectra. It can be seen from the score chart that the group–group are clearly separated, indicating significant differences. The scores of the OPLS-DA were as follows: healthy, CHD, CHD + HTN, CHD + Dep, and CHD + T2DM group: *R*^2^*X* = 0.624, *R*^2^*Y* = 0.697, *Q*^2^ = 0.692; healthy and CHD group: *R*^2^*X* = 0.701, *R*^2^*Y* = 0.978, *Q*^2^ = 0.968; CHD group and CHD + HTN group: *R*^2^*X* = 0.645, *R*^2^*Y* = 0.989, *Q*^2^ = 0.988; CHD group and CHD + Dep group: *R*^2^*X* = 0.623, *R*^2^*Y* = 0.977, *Q*^2^ = 0.971; and CHD group and CHD + T2DM group: *R*^2^*X* = 0.567, *R*^2^*Y* = 0.888, *Q*^2^ = 0.868. These results can be seen in Figure 2. All parameters of the OPLS-DA model (*R*^2^*X*, *R*^2^*Y*, and *Q*^2^*Y*) were positive, indicating that the model was robust.

### 3.4. Identification of Discriminant Metabolites

**Figure 3:**
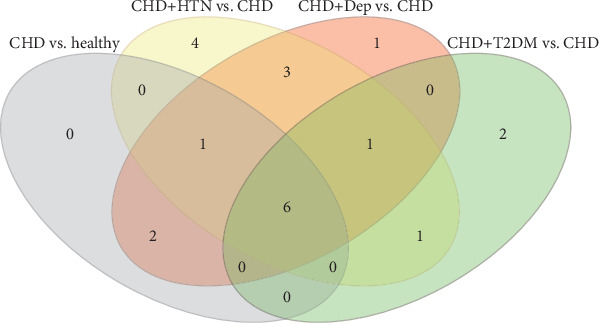
Venn diagram presenting the distribution of metabolites between the healthy and CHD group, CHD and CHD + HTN group, CHD and CHD + Dep group, and CHD and CHD + T2DM group. Note: the numbers in the figure represent the metabolites among different groups.

Metabolites with VIP values > 1.0 in the OPLS-DA and *p* values < 0.05 in the two-tailed Student *t*-tests were deemed as discriminant metabolites. There were 9, 16, 14, and 10 metabolites identified in the healthy and CHD group, CHD and CHD + HTN group, CHD and CHD + Dep group, and CHD and CHD + T2DM group, respectively. These metabolites and metabolic changes are presented in Table 2, whereas the relative distribution of each metabolite in the healthy and CHD group, CHD and CHD + HTN group, CHD and CHD + Dep group, and CHD and CHD + T2DM group is further highlighted in Figure 3. In our study, the numbers in Figure 3 denote the metabolites present in different groups. The numbers within the overlapping regions of the circles in Figure 3 represent the common metabolites, indicating the intersections of the groups. For example, we screened six common metabolites (malic acid, L-alanine, L-lactic acid, sorbitol, D-fructose, and D-maltose) that belong to the healthy and CHD group, the CHD and CHD + HTN group, the CHD and CHD + Dep group, and the CHD and CHD + T2DM group, based on the online Venn diagram tool.

### 3.5. Analyses of Metabolic Pathways

**Figure 4:**
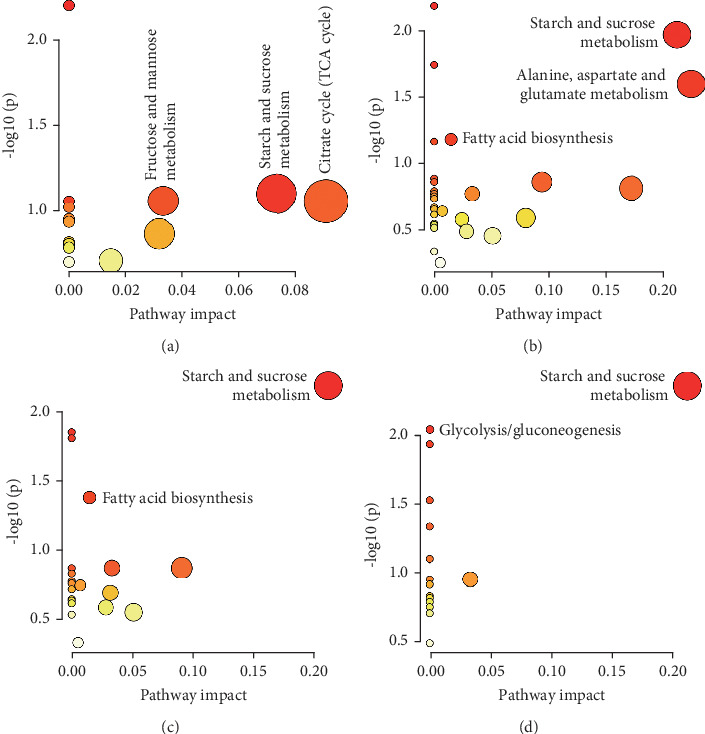
Summary of pathway analysis performed using MetaboAnalyst 5.0. (a) CHD vs. healthy. (b) CHD + HTN vs. CHD. (c) CHD + Dep vs. CHD. (d) CHD + T2DM vs. CHD.

We identified several potentially significant pathways (raw *p* < 0.1, impact > 0) (Table 3). In the healthy and CHD group, the pathways were as follows: starch and sucrose metabolism, fructose and mannose metabolism, and citrate cycle (TCA cycle). Starch and sucrose metabolism; alanine, aspartate, and glutamate metabolism; and fatty acid biosynthesis were discovered in the CHD and CHD + HTN group. Two pathways had the greatest significance in the CHD and CHD + Dep group: starch and sucrose metabolism and fatty acid biosynthesis. Additionally, starch and sucrose metabolism and gluconeogenesis were identified in the CHD and CHD + T2DM group. The detailed results of metabolic pathway analysis are presented in Table 3 and Figure 4.

## 4. Discussion

Currently, although the comorbidities of CHD have attracted increasing academic attention, the mechanisms underlying these comorbidities have not yet been fully elaborated [24–26]. GC–MS-based metabolomics was used in the present study to profile serum metabolic biomarkers in the comorbidities of CHD. Edetic acid, malic acid, L-alanine, L-lactic acid, sorbitol, D-fructose, D-maltose, citric acid, and palmitic acid were identified in the healthy and CHD group. Compared to the CHD group, malic acid, L-alanine, L-lactic acid, sorbitol, D-fructose, D-maltose, palmitic acid, myristic acid, pyroglutamic acid, urea, cholesterol, L-aspartic acid, D-xylitol, glyceric acid, glucose 6-phosphate, and MG (16:0/0:0/0:0) were differentially expressed in the CHD comorbid with HTN group. Fourteen metabolites (edetic acid, malic acid, L-alanine, L-lactic acid, sorbitol, D-fructose, D-maltose, citric acid, palmitic acid, myristic acid, pyroglutamic acid, cholesterol, glucose 6-phosphate, and oleamide) significantly differed in the CHD and CHD + Dep group. In the CHD and CHD + T2DM group, the discriminating metabolites were as follows: malic acid, L-alanine, L-lactic acid, sorbitol, D-fructose, D-maltose, urea, glucose 6-phosphate, D-glucose, and L-threonine. And, these discriminant metabolites were involved in six pathways (Figure 4), mainly pertaining to carbohydrate, lipid, and amino acid metabolism and summarized in Table 3. Herein, we investigated the metabolic changes in each of the comorbidities of CHD and sought to identify potential biomarkers to explore the underlying mechanisms and provide new therapeutic strategies.

### 4.1. The CHD Group Compared to the Healthy Group

In our study, GC–MS-based metabolomics was applied to profile metabolic biomarkers in the serum of 149 CHD patients and 169 healthy controls. The discriminating metabolites were as follows: edetic acid, malic acid, L-alanine, L-lactic acid, sorbitol, D-fructose, D-maltose, citric acid, and palmitic acid. These metabolites were involved in three significantly different pathways related to starch and sucrose metabolism, fructose and mannose metabolism, and TCA cycle.

Previous metabolomic studies have identified multiple metabolites that are associated with CHD [11, 27–29], some of which were corroborated in our study. For example, metabolites involved in the TCA cycle, including malic acid, L-lactic acid, and citric acid, were associated with CHD occurrence in this and other studies. Citric acid is an important component of the TCA cycle that participates in the common metabolic pathways of carbohydrate, fat, and protein. The changes of the malic acid, L-lactic acid, and citric acid indicated that the TCA cycle was disturbed. This conclusion has been confirmed by other studies [30]. The synthesis and metabolism of L-alanine was also disturbed. The synthesis of L-alanine from pyruvate via alanine aminotransferase is directly involved in gluconeogenesis and the alanine–glucose cycle, thus regulating glucose metabolism [30].

### 4.2. The CHD Comorbid With HTN Group Compared to the CHD Group

In the current study, we present the metabolic signatures of 107 CHD + HTN subjects and 149 CHD subjects. These discriminant metabolites were as follows: malic acid, L-alanine, L-lactic acid, sorbitol, D-fructose, D-maltose, palmitic acid, myristic acid, pyroglutamic acid, urea, cholesterol, L-aspartic acid, D-xylitol, glyceric acid, glucose 6-phosphate, and MG (16:0/0:0/0:0), involved in starch and sucrose metabolism; alanine, aspartate, and glutamate metabolism; and fatty acid biosynthesis.

L-Alanine can be derived during the transamination from pyruvate by glutamate. Previous studies have found that glutamate is associated with several risk factors for incident CHD comorbid with HTN [4, 31]. Our study may indicate that the association between alanine and incident CHD comorbid with HTN is driven by glutamate. In line with the alanine results, pyroglutamic acid (also known as 5-oxoproline) is an intermediate in glutathione metabolism. Circulating L-aspartic acid has been associated with decreased BMI, abdominal obesity, and insulin resistance [32]. Our study confirmed that disturbances in L-aspartic acid are related to the incidence of CHD comorbid with HTN.

The metabolism of starch and sucrose begins with D-fructose interacting with a D-glucose. The altered levels of D-fructose, D-maltose, and glucose 6-phosphate demonstrated that starch and sucrose metabolism was affected. Moreover, fatty acid biosynthesis was markedly perturbed in the two groups, suggesting that fatty acid biosynthesis disorders may be involved in the development of CHD-comorbid HTN.

### 4.3. The CHD Comorbid With Dep Group Compared to the CHD Group

Given the long disease course and unsatisfactory prognosis, it is unsurprising that most CHD patients experience Dep. In our study, 126 CHD + Dep subjects and 149 CHD subjects were analyzed and compared. Fourteen metabolites (edetic acid, malic acid, L-alanine, L-lactic acid, sorbitol, D-fructose, D-maltose, citric acid, palmitic acid, myristic acid, pyroglutamic acid, cholesterol, glucose 6-phosphate, and oleamide) associated with starch and sucrose metabolism and fatty acid biosynthesis significantly differed in the two groups. Previous metabolomic studies have identified many metabolites that are associated with Dep [13, 15, 33–36]; some of which were conducted in our lab. In line with a previous study on Dep, L-alanine, L-lactic acid, citric acid, palmitic acid, myristic acid, pyroglutamic acid, cholesterol, glucose 6-phosphate, and oleamide were disturbed. L-Alanine was reportedly increased in heart tissue of chronic unpredictable mild stress rats [37] and thought to be a link between Dep and cardiovascular disease, which was further validated in the CHD comorbid with Dep and CHD groups. Citric acid is a dominant intermediate in the TCA cycle, playing a crucial role in energy metabolism. Fatty acid biosynthesis is known to play an important role in inflammation and oxidative stress. This may explain why, in our study, the levels of cholesterol, oleamide, palmitic acid, and myristic acid were significantly altered in the CHD comorbid with Dep compared with the CHD group.

### 4.4. The CHD Comorbid With T2DM Group Compared to the CHD Group

A total of 58 CHD + T2DM subjects and 149 CHD subjects were compared in our study. The levels of some metabolites involved starch and sucrose metabolism and glycolysis (i.e., malic acid, L-alanine, L-lactic acid, sorbitol, D-fructose, D-maltose, urea, glucose 6-phosphate, D-glucose, and L-threonine) were disturbed. These findings might help shed additional light on why CHD and T2DM commonly co-occur.

L-Alanine is a nonessential amino acid and is synthesized via reductive amination of pyruvate and participates in sugar and acid metabolism. It is also known for playing a central role in the glucose–alanine cycle taking place between the liver and other tissues. This role supports the association between L-alanine and incident CHD and T2DM in our study, which agrees with previous studies that the L-alanine level was associated with incident CHD and T2DM [38–41]. In addition, research indicates that L-alanine supplementation could improve blood glucose levels [42, 43]. Therefore, our findings reinforce previous studies that L-alanine is associated with incident CHD and T2DM. Amino acids have gained the increased attention of researchers, and altered amino acid levels have been identified as markers of risk for incident CHD and T2DM, suggesting that threonine is involved in the development of CHD and T2DM through shared pathways. Disorders of starch and sucrose metabolism and glycolysis in incident CHD and T2DM are unquestionable. Therefore, D-fructose, D-maltose, urea, glucose 6-phosphate, and D-glucose are the metabolites with the strongest evidence for a connection with CHD and T2DM.

To some extent, our findings may offer several practical implications for primary care physicians. Firstly, the identification of potential biomarkers in the comorbidities of CHD may provide a basis for targeted screening and early intervention strategies. By incorporating these potential biomarkers into routine patient assessments, physicians can more effectively identify individuals at higher risk, allowing for earlier diagnosis and treatment. Secondly, our study's findings may guide clinical decision-making. Primary care physicians could utilize this evidence to make informed choices regarding patient management, potentially improving outcomes through more effective treatment plans.

### 4.5. Strengths and Limitations

Our study systematically studied changes of metabolites in the comorbidities of CHD through metabolomic methods. These findings may be used to offer several practical implications for primary care physicians and help to explore the underlying mechanisms of the comorbidities of CHD. However, several limitations must be mentioned. First, a single GC–MS-based metabolomic method was used in our study and could be improved by also using nuclear magnetic resonance and LC–MS or combined with proteomics, genomics, and other multiple omics methods to further validate our findings. Second, a larger sample size (i.e., CHD comorbid with HTN group, CHD comorbid with Dep group, and CHD comorbid with T2DM group) should be collected. Finally, the diagnostic value of the potential biomarkers should be validated with setting up internal and external validation sets and multicenter in clinical practice.

## 5. Conclusion

In the current study, a GC–MS-based profiling of CHD, HTN-comorbid CHD, Dep-comorbid CHD, and T2DM-comorbid CHD was employed to systemically discover relevant biomarkers. Our study provides a panoramic and systematic view of metabolic alterations in CHD comorbidity that correlate with amino acid, lipid, and energy metabolism, providing predictive information for CHD comorbidity and enhancing our understanding of the CHD comorbidity.

## Figures and Tables

**Table 1:** Clinical characteristics of the subjects.

**Variables**	**Healthy (** **n** = 169**)**	**CHD (** **n** = 149**)**	**p** ** value** ^a^	**CHD + HTN (** **n** = 107**)**	**p** ** value** ^b^	**CHD + Dep (** **n** = 126**)**	**p** ** value** ^c^	**CHD + T2DM (** **n** = 58**)**	**p** ** value** ^d^
Age (years)	59.69 ± 6.69	60.32 ± 11.90	0.565	60.60 ± 11.54	0.853	62.39 ± 10.74	0.135	62.47 ± 11.28	0.239
BMI (kg/m^2^)	23.54 ± 3.27	24.21 ± 3.03	0.062	23.77 ± 3.12	0.258	23.59 ± 3.14	0.099	24.01 ± 3.29	0.677
Gender (M/F, *n*)	91/78	86/63	0.488	59/48	0.681	60/66	0.095	28/30	0.22
Smoking (*n*, %)	58 (34.3)	55 (36.9)	0.63	41 (38.3)	0.819	43 (34.1)	0.631	19 (32.8)	0.575
Drinking (*n*, %)	55 (32.5)	57 (38.3)	0.287	41 (38.3)	0.992	39 (31)	0.206	20 (34.5)	0.614

Abbreviations: BMI, body mass index; CHD, coronary heart disease; CHD + Dep, CHD with depression; CHD + HTN, CHD with hypertension; CHD + T2DM, CHD with Type 2 diabetes mellitus.

^a^CHD vs. healthy.

^b^CHD + Dep vs. CHD.

^c^CHD + Dep vs. CHD.

^d^CHD + T2DM vs. CHD.

**Table 2:** List of metabolites with changes between healthy and CHD group, CHD and CHD + HTN group, CHD and CHD + Dep group, and CHD and CHD + T2DM group.

**Metabolites**	**HMDB**	**CHD vs. healthy**	**CHD + HTN vs. CHD**	**CHD + Dep vs. CHD**	**CHD + T2DM vs. CHD**
		**VIP**	**VIP**	**VIP**	**VIP**
Edetic acid	HMDB0015109	4.618		4.196	
Malic acid	HMDB0000744	2.903	2.451	2.981	3.839
L-Alanine	HMDB0000161	1.983	1.584	1.88	1.73
L-Lactic acid	HMDB0000190	1.952	2.456	1.655	1.813
Sorbitol	HMDB0000247	1.642	1.504	1.701	2.21
D-Fructose	HMDB0000660	1.526	1.61	1.632	2.047
D-Maltose	HMDB0000163	1.373	1.624	1.402	3.299
Citric acid	HMDB0000094	1.062		1.023	
Palmitic acid	HMDB0000220	1.013	1.135	1.005	
Myristic acid	HMDB0000806		2.657	1.004	
Pyroglutamic acid	HMDB0000267		2.615	1.152	
Urea	HMDB0000294		1.466		1.097
Cholesterol	HMDB0000067		1.305	1.051	
L-Aspartic acid	HMDB0000191		1.272		
D-Xylitol	HMDB0002917		1.182		
Glyceric acid	HMDB0000139		1.058		
Glucose 6-phosphate	HMDB0001401		1.058	1.076	1.75
MG (16:0/0:0/0:0)	HMDB0011564		1.028		
Oleamide	HMDB0002117			1.065	
D-Glucose	HMDB0000122				1.523
L-Threonine	HMDB0000167				1.09

**Table 3:** Pathway analysis performed using MetaboAnalyst 5.0 software.

**Pathway name**	**CHD vs. healthy**		**CHD + HTN vs. CHD**		**CHD + Dep vs. CHD**		**CHD + T2DM vs. CHD**	
	**Raw ** **p**	**Impact**	**Raw ** **p**	**Impact**	**Raw ** **p**	**Impact**	**Raw ** **p**	**Impact**
Starch and sucrose metabolism	0.0787	0.0731	0.0107	0.212	0.00659	0.212	0.00437	0.212
Fructose and mannose metabolism	0.0871	0.0331						
TCA cycle	0.0871	0.0904						
Alanine, aspartate, and glutamate metabolism			0.0251	0.224				
Fatty acid biosynthesis			0.0650	0.0147	0.0416	0.0147		
Glycolysis/gluconeogenesis							0.00906	0.00021

## Data Availability

The raw data of this manuscript will be made available by the authors.

## Supporting Information

Supporting Information 1 Details of the definitions of *R*^2^*X*, *R*^2^*Y*, and *Q*^2^*Y*, as well as the metabolomic samples and data processing, can be found in the supporting information under Section 2.5.

Supporting Information 2 Table S1: Odds ratios from logistic regression of clinical characteristics of the subjects among the following comparisons: CHD vs. healthy, CHD + Dep vs. CHD, CHD + HTN vs. CHD, and CHD + T2DM vs. CHD.
