# Supplementary material for: Metabolomic Profiling Reveals Biomarkers in Coronary Heart Disease Comorbidity
Source: J Diabetes Res. 2024 Dec 19;2024:8559677. doi: 10.1155/jdr/8559677 (PMC11671664; doi:10.1155/jdr/8559677)
Supplement: Supporting Information 1 — Details of the definitions of R2X, R2Y, and Q2Y, as well as the metabolomic samples and data processing, can be found in the supporting information under Section 2.5. [file 8559677.f1.docx]

**Supporting Information**

**Metabolomics profiling reveals biomarkers in coronary heart disease** **comorbidity**

Chunmei Geng^1^, Benhui Liang^2^, Zihan Kong^3^, Lei Feng^4^, Jianhua Wang^3^, Qingying Si^5^, Pei Jiang^3^*

^1^ Department of Pharmacy, Tianjin First Central Hospital, Nankai University, Tianjin, China

^2^ Department of Cardiology, Xiangya Hospital, Central South University, Changsha, Hunan, China

^3^ Translational Pharmaceutical Laboratory, Jining First People’s Hospital, Shandong First Medical University, Jining, Shandong, China

^4^ Department of Neurosurgery, Jining First People’s Hospital, Shandong First Medical University, Jining, Shandong, China

^5^ Department of Endocrinology, Tengzhou Central People's Hospital, Tengzhou, Shandong, China

***2.5. Statistical analyses***

Demographic and clinical characteristics of the subjects were evaluated with the Student’s *t*-test (for normally distributed continuous variables), the Mann–Whitney test (for non-normally distributed continuous variables) and logistic regression analysis. Categorical variables were displayed as numbers (percentage) of subjects within each group and were compared using the χ2 test. The above analyses were performed via SPSS 23.0 software (SPSS Inc, Chicago, IL, USA).

The primary GC–MS data were processed using Agilent Unknowns analysis software and Masshunter quantitative analysis software (Agilent Technologies, CA, USA). This process enabled peak extraction, removal of peaks with a signal-to-noise ratio< 3, and peak deconvolution, alignment, and data reduction to produce a list of m/z and RT pairs, with the corresponding intensities for all detected peaks from each data file in the dataset^[20, 21]^. The resulting table was exported into Excel^TM^ (Microsoft, Redmond, WA, United States), and the normalized peak area percentages were used as the percentage of corresponding intensities of each peak/total peak area. Center scaling, unit variance scaling, and pareto scaling are commonly used to perform the normalization data. In our study, we adopted the pareto scaling. Too many missing values will cause difficulties for downstream analysis. There are several different methods for this purpose, such as replace by a small values, mean/median, k-nearest neighbor (KNN), probabilistic principal components analysis (PPCA), Bayesian PCA (BPCA) method, and singular value decomposition (SVD) method to impute the missing values ^[22,23]^. In our work, the default method replaces all the missing values with small values (the half of the minimum positive values in the original data) assuming to be the detection limit, and the data were not transformed. The resulting three-dimensional dataset including peak index (RT–m/z pairs), sample names (observations), and normalized peak area percentages was imported into SIMCA-P 14.0 (Umetrics, Umea, Sweden) for statistical analyses. SIMCA-P 14.0 software (Umetrics, Umea, Sweden) was used to perform principal component analysis (PCA), partial least squares-discriminant analysis (PLS-DA), and orthogonal partial least squares-discriminant analysis (OPLS-DA) of the healthy group and the CHD group, the CHD group and the CHD+HTN group, the CHD group and the CHD+Dep group, the CHD group and the CHD+T2DM group, to select the significant variables that were responsible for group separation. R^2^X, R^2^Y, Q^2^Y, and variable importance in projection (VIP) values are important quality parameters for evaluating the performance of OPLS-DA models.

R^2^X, this is the model's explanatory power for the X matrix (sample data), which is the percentage of the data variance that the model can explain. The closer the R^2^X value is to 1, the stronger the model's explanatory power for the data. In principal component analysis (PCA) analysis, an R^2^X greater than 0.4 is generally considered good, but in partial least squares-discriminant analysis (PLS-DA) and OPLS-DA analyses, the importance of R^2^X is less than that of R^2^Y and Q^2^Y.

R^2^Y, this is the model's explanatory power for the Y matrix (response variables or classification information). The closer the R^2^Y value is to 1, the more classification information the model can explain, meaning that the model can better distinguish between different sample groups. R^2^Y is an important indicator for assessing the model's interpretability.

Q^2^Y, this is calculated through cross-validation and is used to assess the model's predictive power. The closer the Q^2^Y value is to 1, the stronger the model's predictive power and the more reliable the model is. Generally, a Q^2^Y value greater than 0.5 is considered an indicator of an effective model, while a Q^2^Y value greater than 0.9 is considered an excellent model.

VIP value, this is a measure of how much a variable contributes to the separation between the groups in the model. It is calculated by summing the squared weights of the variable across all components of the OPLS-DA model. In the context of metabolomics field, metabolites with VIP values greater than 1.0 in the OPLS-DA are considered to be significant contributors to the model's ability to distinguish between different groups or classes. They may be potential biomarkers or play a key role in the biological processes that distinguish one group from another.

The two-tailed Student’s t-tests were performed using SPSS 23.0 to further test the differences between the two groups. Metabolites with variable importance in projection (VIP) values > 1.0 in the OPLS-DA analysis and p values< 0.05 in the two-tailed Student’s *t*-tests were considered potential discriminant metabolites. Metabolic pathways, as studied through metabolomics, have a wide range of applications, from identifying disease biomarkers to understanding the effects of dietary interventions and drug actions. Therefore, understanding these pathways is crucial for uncovering disease mechanisms, identifying therapeutic targets, and developing strategies for early diagnosis and treatment. Metabolic pathways analysis is often conducted with the use of tools such as KEGG (Kyoto Encyclopedia of Genes and Genomes), MetaboAnalyst, Reactome, BioCyc, and MetaCyc, which provide comprehensive databases of these pathways across different organisms. In tools like MetaboAnalyst, the Pathway Impact Value is often used in conjunction with p-values to assess the significance of metabolic pathways. Pathways with p-values less than 0.1 (indicating statistical significance) and impact values greater than 0, as these pathways may play significant roles in biology or pathology. However, it is important to note that there is no fixed threshold for impact values; they are relative. Generally, a larger impact value suggests a more significant role in the metabolic pathway. In our study, the finalized set of discriminant metabolites was imported into MetaboAnalyst 5.0 (http://www.metaboanalyst.ca) for metabolic pathway analysis, and pathways with p-values <0.1 and impact values > 0 were defined as significant, contributing to the biochemical interpretation of the metabolites.Venn diagrams are indeed a useful tool in metabolomics, helping to identify common and unique metabolites by comparing different groups. They clearly show the relationships and intersections between different sets of data. In our study, we aimed to discover common metabolites among the following groups: Healthy and CHD, CHD and CHD+HTN, CHD and CHD+Dep, and CHD and CHD+T2DM, using an online Venn diagram tool (<http://www.omicshare.com/tools>).
