# Supplementary material for: Metabolomic Profiling Reveals Biomarkers in Coronary Heart Disease Comorbidity
Source: J Diabetes Res. 2024 Dec 19;2024:8559677. doi: 10.1155/jdr/8559677 (PMC11671664; doi:10.1155/jdr/8559677)
Supplement: Supporting Information 2 — Table S1: Odds ratios from logistic regression of clinical characteristics of the subjects among the following comparisons: CHD vs. healthy, CHD + Dep vs. CHD, CHD + HTN vs. CHD, and CHD + T2DM vs. CHD. [file 8559677.f2.docx]

Table S1. Odds ratios from logistic regression of clinical characteristics of the subjects among the following comparisons: CHD vs. Healthy, CHD+Dep vs. CHD, CHD+HTN vs. CHD, and CHD+T2DM vs. CHD

| Variables | OR, 95%CI | p-value^a^ | OR, 95%CI | p-value^b^ | OR, 95%CI | p-value^c^ | OR, 95%CI | p-value^d^ |
| --- | --- | --- | --- | --- | --- | --- | --- | --- |
| Age (years) | 1.009 (0.985-1.033) | 0.467 | 0.998 (0.976-1.020) | 0.838 | 0.986 (0.965-1.008) | 0.206 | 0.983 (0.956-1.010) | 0.212 |
| BMI (kg/m2) | 1.071 (0.998-1.150) | 0.058 | 1.051 (0.966-1.142) | 0.251 | 1.071(0.990-1.161) | 0.088 | 1.031 (0.933-1.140) | 0.548 |
| Gender; male vs. female | 0.958 (0.593-1.550) | 0.863 | 0.782 (0.389-1.577) | 0.493 | 0.689 (0.370-1.285) | 0.241 | 0.591 (0.249-1.398) | 0.231 |
| Smoking; non-smoking vs. smoking | 1.076 (0.598-1.940) | 0.805 | 1.136 (0.564-2.292) | 0.719 | 1.193(0.638-2.231) | 0.581 | 0.906 (0.409-2.006) | 0.808 |
| Drinking; non-drinking vs. drinking | 0.717 (0.390-1.318) | 0.284 | 1.048 (0.493-2.290) | 0.901 | 0.831(0.427-1.617) | 0.586 | 1.391 (0.534-3.618) | 0.499 |

^a^CHD vs. Healthy, ^b^CHD+Dep vs. CHD, ^c^CHD+Dep vs. CHD, ^d^CHD+T2DM vs. CHD. BMI, body mass index; CHD, coronary heart disease; CHD+HTN, CHD with hypertension; CHD+Dep, CHD with depression; CHD+T2DM, CHD with Type 2 diabetes mellitus
